# D,L-Methadone enhances the cytotoxic activity of standard chemotherapeutic agents on pediatric rhabdomyosarcoma

**DOI:** 10.1007/s00432-022-03945-y

**Published:** 2022-02-19

**Authors:** Cristian Urla, Irene Corteletti, Ann-Sophie Raible, Rupert Handgretinger, Jörg Fuchs, Steven W. Warmann, Evi Schmid

**Affiliations:** 1 Department of Pediatric Surgery and Pediatric Urology, University Children’s Hospital of Tuebingen, Hoppe-Seyler-Str. 3, 72076 Tuebingen, Germany; 2 Department of Molecular Medicine, University of Padova, Via Gabelli 63, 35121 Padova, Italy; 3 Department of Pediatric Hematology and Oncology, University Children’s Hospital of Tuebingen, Hoppe-Seyler-Str. 1, 72076 Tuebingen, Germany

**Keywords:** Methadone, Chemotherapy, Cytotoxicity, Pediatric, Rhabdomyosarcoma

## Abstract

**Purpose:**

In advanced tumor stages, pediatric rhabdomyosarcoma (RMS) shows an intrinsic resistance to standard chemotherapy, which is associated with a dismal prognosis. Alternative therapeutic approaches and optimization of already existent treatment protocols are urgently needed in these conditions. The µ-opioid receptor (OPRM1) agonist, D,L-methadone is frequently used for analgesia in oncological patients. Recent evidence has shown that D,L-methadone in combination with chemotherapeutic agents may enhance their cytotoxic effect against cancer cells. There are no related data in pediatric rhabdomyosarcoma (RMS).

**Methods:**

Antitumor effects of combined D,L-methadone and doxorubicin, carboplatin, and vincristine on RMS cell lines RD and RH30 were analyzed using following outcome data: expression of the OPRM1 receptor (Western blot), cell growth inhibition (MTT assay), cell migration (wound-healing assay), apoptosis induction (caspase-3/7 assay), and reactive oxygen species (ROS) production (flow cytometry).

**Results:**

In both cell lines, OPRM1 expression was significantly increased after combined treatment of D,L-methadone with all three cytotoxic drugs tested, which resulted in suppression of tumor cell growth and increase of apoptosis rates. These effects were mediated by increased ROS production and up-regulation of caspase-3/7 activity. Doxorubicin combined with D,L-methadone significantly reduced cell migration in both cell lines. Carboplatin or vincristine in combination with D,L-methadone had only an impact on cell migration in RH30 cells.

**Conclusions:**

This new therapeutic approach in RMS provides strong antitumor effects in vitro. The combination of standard chemotherapy and D,L-methadone requires further investigation. Especially advanced tumors with a limited effectiveness of conventional treatment regimens seem a potential target of this approach.

**Supplementary Information:**

The online version contains supplementary material available at 10.1007/s00432-022-03945-y.

## Introduction

Rhabdomyosarcoma (RMS) is the most common soft tissue sarcoma, accounting for 5% of all cases of pediatric solid tumors (Dasgupta et al. 2016). The two main histopathological subtypes of this malignancy in children are embryonal (RME) and alveolar (RMA) (Seitz et al. 2007).

The prognosis of children with RMS has dramatically improved since introduction of multimodal treatment regimens. Cure rates have increased from 25% in the early 70s to approximately 70% in more recent years (Dasgupta et al. 2016). A major role in developing new therapeutic strategies has been carried out by cooperative clinical trial groups in Europe and North America (Dantonello et al. 2008; Fuchs et al. 2018; Meza et al. 2006; Ferrari et al. 2003). In all trials, the patients are treated according to the risk group they were assigned. Chemotherapy remained an essential component of the multimodal treatment. Within CWS trials, standard-risk patients receive ifosfamide, vincristine and actinomycin-D. Very high-risk patients are additionally treated with doxorubicin. Patients with relapse receive a second-line chemotherapy including topotecan, etoposide, carboplatin, and cyclophosphamide Klingebiel et al. 1998; Längler et al. 2002).

Local tumor recurrence, development of multidrug resistance, and metastatic disease are the most challenging issues, which treating physicians are confronted with (Seitz et al. 2007). Despite aggressive multimodal treatment, the prognosis of children suffering from advanced stages of disease is dismal as only 25% of them are expected to be free of disease 3 years after diagnosis (Dasgupta et al. 2016). Therefore, the development of new therapeutic strategies is of critical importance.

Methadone is a synthetic opioid which is usually used as a racemic mixture (D,L-methadone) for pain management. It can be applied as a second-line analgetic in patients with cancer pain, especially when effects of other opioids decrease or when the patients present severe side effects against first-line opioids (Michalska et al. 2018).

Recent preclinical evidence has shown that D,L-methadone may increase the effects of chemotherapy in different tumor cell populations and is, therefore, discussed as being a sensitizer for chemotherapy (Friesen et al. 2008, 2014; Heusch and Maneckjee 1999; Bortsov et al. 2012; Michalska et al. 2018). Friesen et al. found that D,L-methadone inhibits proliferation and induces cell apoptosis through caspase-3/9 activation and down-regulation of Bcl-x_L_ as well as X chromosome-linked inhibitor of apoptosis in leukemia cells (Friesen et al. 2008). Furthermore, they observed that D,L-methadone induced cell death not only in chemo- and apoptosis-sensitive leukemia cells but also in doxorubicin-resistant, multidrug-resistant as well as in apoptosis-resistant leukemia cells (Friesen et al. 2008). Landgraf et al. have also reported that D,L-methadone significantly enhance the toxicity of doxorubicin and cisplatin in HLaC78 cells (larynx carcinoma) (Landgraf et al. 2019). Stadlbauer et al. demonstrated that co-incubation of doxorubicin with D,L-methadone determines a dose-dependent increase in the apoptosis rate up to 88% in PC-3 cells (prostate cancer) (Stadlbauer et al. 2017).

There are no systematical analyses in the literature addressing the influence of D,L-methadone in combination with chemotherapeutic drugs on pediatric RMS cells. Therefore, the aim of the present study was to investigate if D,L-methadone in combination with cytotoxic drugs (doxorubicin/carboplatin/vincristine) influences the cell viability, migration, apoptosis induction, and signaling pathway in pediatric RMS cell lines.

## Materials and methods

### Cell lines and culture conditions

The RME cell line RD (ATCC, Manassas, VA, USA), the RMA cell line RH30 (DSMZ, Braunschweig, Germany), and the human skeletal muscle cells (SkMC; Merck, Taufkirchen, Germany) were routinely cultured in DMEM medium with L-glutamine (Biochrom, Berlin, Germany) supplemented with 10% fetal bovine serum (FBS, Biochrom, Berlin, Germany), and 1% penicillin/streptomycin (Biochrom, Berlin, Germany) in a humidified atmosphere containing 5% CO_2_ at 37 °C. All cells were tested to be mycoplasma negative (Mycoalert, Lonza, Basel, Switzerland).

### Cell viability assay

Cells were seeded in 96-well plates at a density of 8 × 10^3^ cells per well in triplicate. After overnight adherence of the cells and 72 h treatment with D,L-methadone as single agent or in combination with chemotherapeutic drugs (doxorubicin/carboplatin/vincristine), cell viability was determined using a colorimetric MTT assay (Merck, Darmstadt, Germany) measuring the reduction of tetrazolium salts to formazan derivatives by functional mitochondria. Lysis buffer (DMSO, SDS, acid) was added to solubilize the blue MTT-formazan product. The assay was performed as originally described by Mosmann et al. (1983). Absorbance was measured at 570 nm.

### Flow cytometry analysis for quantification of apoptosis

For flow cytometry-based apoptosis measurements, RD and RH30 cells were seeded in 6-well plates at a density of 1 × 10^5^ cells per well. Following attachment, the cells were treated with D,L-methadone alone or in combination with chemotherapeutic drugs for 72 h. After incubation cells were collected, washed twice with PBS and resuspended in 1 × Annexin-binding-buffer (EXBIO Praha, Czech Republic). To analyze the cells, 3.5 µl Annexin-V-APC (ImmunoTools, Friesoythe, Germany) and 3.5 µl Propidium Iodide (PI; Merck, Taufkirchen, Germany) were added to 100 µl cell suspension and incubated at RT for 30 min in the dark. Thereafter, 200 µl Annexin-V binding buffer was added and apoptosis was analyzed using a BD Canto II flow cytometer (Becton Dickinson, Heidelberg, Germany).

### Wound-healing assay

Cells were seeded in 6-well plates at a density of 8 × 10^5^ cells/well in triplicates. Subsequently, after 24 h, an orthogonal scratch of about 1 mm width was performed using a pipette tip (10–100 µl). To prevent the detached cells to reattach to the bottom, the cells were washed with PBS, treated with chemotherapeutic drugs and/or D,L-methadone, and photographed as 0 h control. After 24 h, the cells were photographed again and the extent to which the gap width had narrowed was evaluated using the AxioVision software (Carl Zeiss Microscopy, Oberkochen, Germany). The mean values of 3 photos/well were calculated and compared.

### Colony-forming assay

RD and RH30 cells were plated in 6-well plates at 500 cells per well in 2 ml of media. After attachment cells were treated with the chemotherapeutic drugs and/or D,L-methadone for 72 h in a humidified atmosphere of 37 °C and 5% CO_2_. After 72 h, the cells were washed with PBS and fresh medium without drugs was added. The colonies grew 7–10 days before being fixed with 80% methanol for 5 min and stained with 1% (w/v) crystal violet for 30 min for RD cells and 120 min for RH30 cells. The number of colonies (> 50 cells) was counted microscopically (Schmid et al. 2016).

### Measurement of reactive oxygen species (ROS) production

For flow cytometry-based ROS measurements, RD and RH30 cells were seeded in 6-well plates at a density of 1 × 10^5^ cells/well. Following attachment, the cells were treated either with D,L-methadone alone or in combination with the chemotherapeutic drugs for 24 h or with H_2_O_2_ (1:30 previously diluted in DMEM medium with supplements) for 30 min as positive control. ROS measurement was monitored using DCFH-DA (Merck, Taufkirchen, Germany), which was added to the cells (1 µl/well) after treatment for 30 min at 37 °C. Thereafter, attached cells were collected and washed twice with DMEM. 7-AAD (5 µl; Thermo Fisher Scientific, Darmstadt, Germany) was added to the cell suspension and incubated for 15 min at RT in the dark to exclude nonviable from viable cells. Finally, 100 µl DMEM was added and ROS production was analyzed on a BD CANTO II flow cytometer (Becton Dickinson, Heidelberg, Germany).

### Caspase 3/7 activity assay

Measurements of caspase activities in cells were performed using the commercially available Caspase-Glo 3/7 Assay (Promega, Madison, WI) according to the protocol provided by the manufacturer. Briefly, RD and RH30 cells were cultured at 1 × 10^4^ cells (RD) and at 7.5 × 10^3^ cells (RH30) per well in black 96-well plates. Twenty-four hours after seeding, the cells were treated with either D, L-methadone in the presence or absence of chemotherapeutic drugs for 24 h.

### Western blotting

To analyze the expression levels of the µ-opioid receptor OPRM1 under treatment with doxorubicin, carboplatin, and vincristine, whole cell extracts from RD, RH30, and SkMC were prepared using RIPA buffer (Cell Signaling Technology, Inc.) and incubated on ice for 30 min. The whole cell extracts were centrifuged at 4 °C, 14,000×*g* for 20 min, and the protein concentration of the supernatant was determined by Bradford assay (Bio-Rad laboratories GmbH, Feldkirchen, Germany). Lysates (30 µg) were subjected to 10% SDS-PAGE, and proteins transferred to a nitrocellulose membrane (VWR International GmbH), and the membranes were blocked for 1 h at room temperature with 10% non-fat dried milk (Carl Roth GmbH & Co. KG) in TBS-T. For immunoblotting, the membranes were incubated over night at 4 ºC with an antibody directed against OPRM1 (1:200; Thermo Scientific Technology, #PA5-26138). Anti-GAPDH antibody (1∶1,000; Cell Signaling Technology, Inc., #2118S) was used as a loading control. After incubation for 1 h at room temperature with a secondary anti-rabbit IgG antibody conjugated to horseradish peroxidase (1∶3,000; Cell signaling Technology, Inc., #7074S), the proteins were visualized by the WesternSure^®^ PREMIUM Chemiluminescent Substrate (LI-COR Biosciences). Specific bands were quantified with the Odyssey Fc Imaging System (LI-COR Biosciences).

### Evaluation of drug interaction

To analyze the additive or synergistic effect of D,L-methadone and the chemotherapy agents, the coefficient of drug interaction (CDI) was calculated. This is based on the Bliss independence model (Foucquier and Guedj 2015). The CDI is calculated as follows: CDI = (A + B − A × B)/AB. AB is the ratio of absorption of the combination drugs to the control; A or B represents the ratio of the single-drug group to the control group. CDI values < 1, = 1, or > 1 indicate that the drugs are synergistic, additive, or antagonistic, respectively.

### Statistics

Data analysis was performed using the software GraphPad Prism 8 (GraphPad Software Inc., La Jolla, USA). Data are provided as means ± SEM, *n* represents the number of independent experiments. All data were tested for significance using unpaired Student *t* test (Welch’s correction) or ANOVA (Dunnett or Bonferroni correction). Only results with *p* < 0.05 were considered statistically significant.

## Results

### Expression pattern of µ-opioid receptor (OPRM1) in RMS cells

**Fig. 1:**
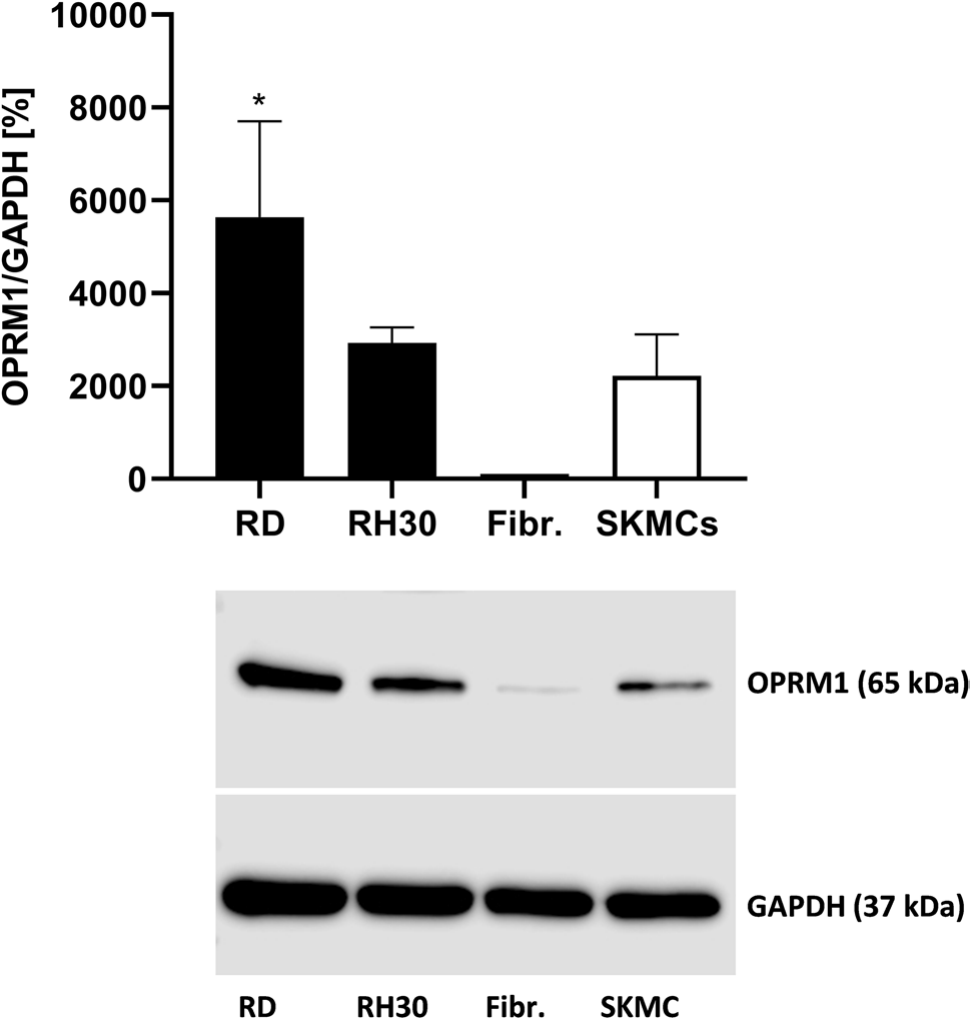
Expression profile of µ-opioid receptor (OPRM1) in RMS cell lines. Protein lysates were obtained and the OPRM1 expression was detected and quantified by Western blot analysis. GAPDH served as housekeeping gene. RD: embryonal RMS cell line; RH30: alveolar RMS cell line; SkMC: human skeletal muscle cells. *(*p* < 0.05), indicate statistical significance to SkMC (ANOVA, Dunnett correction)

An OPRM1 profile for the RMS cell lines RD (RME), RH30 (RMA), SkMC (skeletal muscle cells), and fibroblasts were established. Western blot analysis showed that the OPRM1 is present in detectable amounts in all examined cell lines, although there is a great variability in the degree of expression. RD cells exhibit significant higher receptor expression compared to SkMC and Fibroblasts as shown in Fig. 1.

### Effects of D,L-methadone on viability of RMS cells

**Fig. 2:**
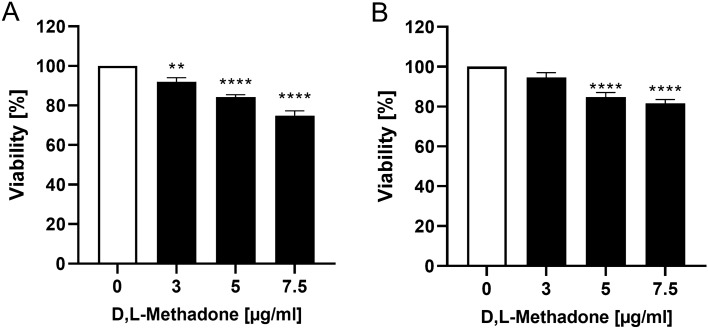
MTT assay. Effects of different concentrations of D,L-methadone on viability of RMS cells. Arithmetic means ± SEM (*n* = 6) of the relative number of viable RD (**A**) and RH30 (**B**) cells after 72 h incubation in the presence of different concentration of D,L-methadone. **(*p* < 0.01), ****(*p* < 0.0001) indicate statistical significance to untreated control (ANOVA, Dunnett correction)

The response of RMS cells to different concentrations of D,L-methadone is presented in Fig. 2 as percentage value of viable cells, compared to untreated sample (set at 100%). In both RMS cell lines, the cell viability was significantly reduced to approximately 80% by treatment with 5 µg/ml and 7.5 µg/ml D,L-methadone. No difference between histological subtypes was observed.

### Effects of D,L-methadone on colony-forming capacity of RMS cells

**Fig. 3:**
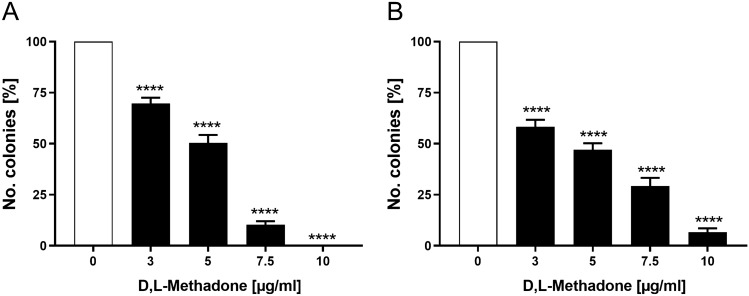
Effects of D,L-methadone on colony-forming capacity of RMS cells. Arithmetic means ± SEM (*n* = 3) of the relative numbers of evolving clones of RD (**A**) and RH30 (**B**) cells following incubation for 72 h in the absence (white bars) and presence of various concentrations of D,L-methadone (black bars). ****(*p* < 0.0001) indicate statistically significant difference to untreated control (ANOVA, Dunnett correction)

To investigate whether D,L-methadone exerts an effect on colony-forming capacity of RMS cells, a colony-forming assay was performed. As depicted in Fig. 3, in both cell lines, the colony-forming capacity was significantly reduced starting from a concentration of 3 µg/ml D,L-methadone. The relative number of colonies was reduced to approximately 10% in the presence of 10 µg/ml D,L-methadone.

### Effects of D,L-methadone on migration of RMS cells

**Fig. 4:**
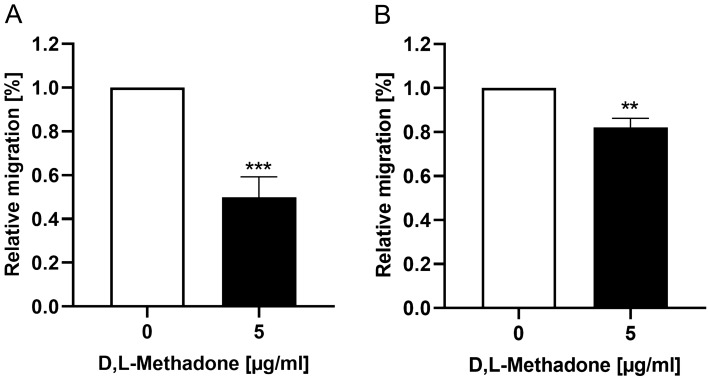
Effects of D,L-methadone on migration ability of RMS cells. Arithmetic means ± SEM (n = 6) of the migration values of RD (**A**) and RH30 cells (**B**) in the absence (white bar) and presence of 5 µg/ml D,L-methadone (black bars). **(*p* < 0.01), ***(*p* < 0.001) indicates statistically significant difference to untreated control (ANOVA, Dunnett correction)

To analyze the influence of D,L-methadone on migration capacity of RMS cells, a wound-healing assay was performed. The migration ability is expressed as percentage of the closure of the initial wound area during 24 h in relation to untreated control (normalized to 100%). As demonstrated in the Fig. 4, treatment of the cells (RD, RH30) with D,L-methadone significantly inhibited cell motility, expressed as larger wound width compared to untreated cells.

### Effects of doxorubicin, carboplatin, and vincristine on the expression of OPRM1 in RMS cells

It has been reported that prolonged exposure of leukemia and glioblastoma cells to doxorubicin lead to an increased expression levels of OPRM1 on these cells (Friesen et al. 2013, 2014). To verify whether this issue is reproducible in RMS cells, we performed Western blot analyses for the semi-quantitative analysis of OPRM1 expression at different time points after treatment with doxorubicin, carboplatin, and vincristine.

**Fig. 5:**
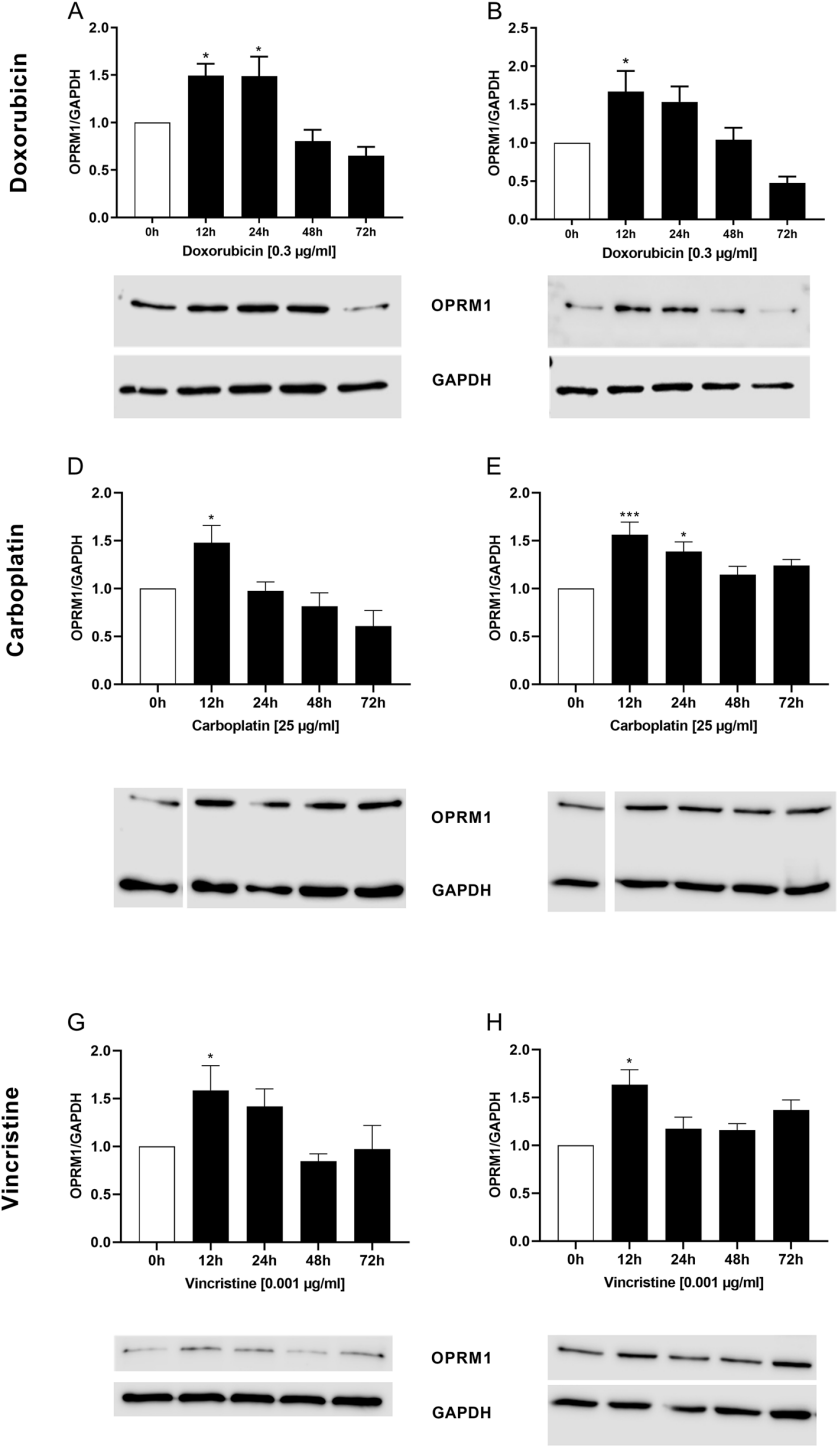
Effects of chemotherapeutic drugs on the expression of OPRM1 in RMS cells. Arithmetic means ± SEM (*n* = 5) of time-dependent expression and abundance levels of OPRM1 in RD (left) and RH30 (right) cells after treatment with doxorubicin (**A**, **B**), carboplatin (**C**, **D**), and vincristine (**D**, **E**). *(*p* < 0.05), ***(*p* < 0.001) indicate statistically significant difference compared to control (ANOVA, Dunnett correction); GAPDH was used as loading control

As shown in Fig. 5, the treatment with doxorubicin led to a significant increase of OPRM1 expression after 12 h (RD + RH30) and 24 h (RD). The exposure of the cells to carboplatin or vincristine also determined a significant increase of receptor expression, the highest significant level being reached after 12 h in both cell lines.

### Effects of combined treatment D,L-methadone and chemotherapy on the viability of RMS cells

Preclinical evidence has demonstrated that D,L-methadone may enhance the cytotoxicity of chemotherapeutic drugs, depending on the drug used and the cell line tested (Landgraf et al. 2019). Therefore, we performed an additional set of experiments to test, whether D,L-methadone enhances the cytotoxic effects of doxorubicin, carboplatin, and vincristine on RMS cell lines. A concentration of 5 µg/ml D,L-methadone was used, as higher concentrations determined a marked cytotoxic effect.

**Fig. 6:**
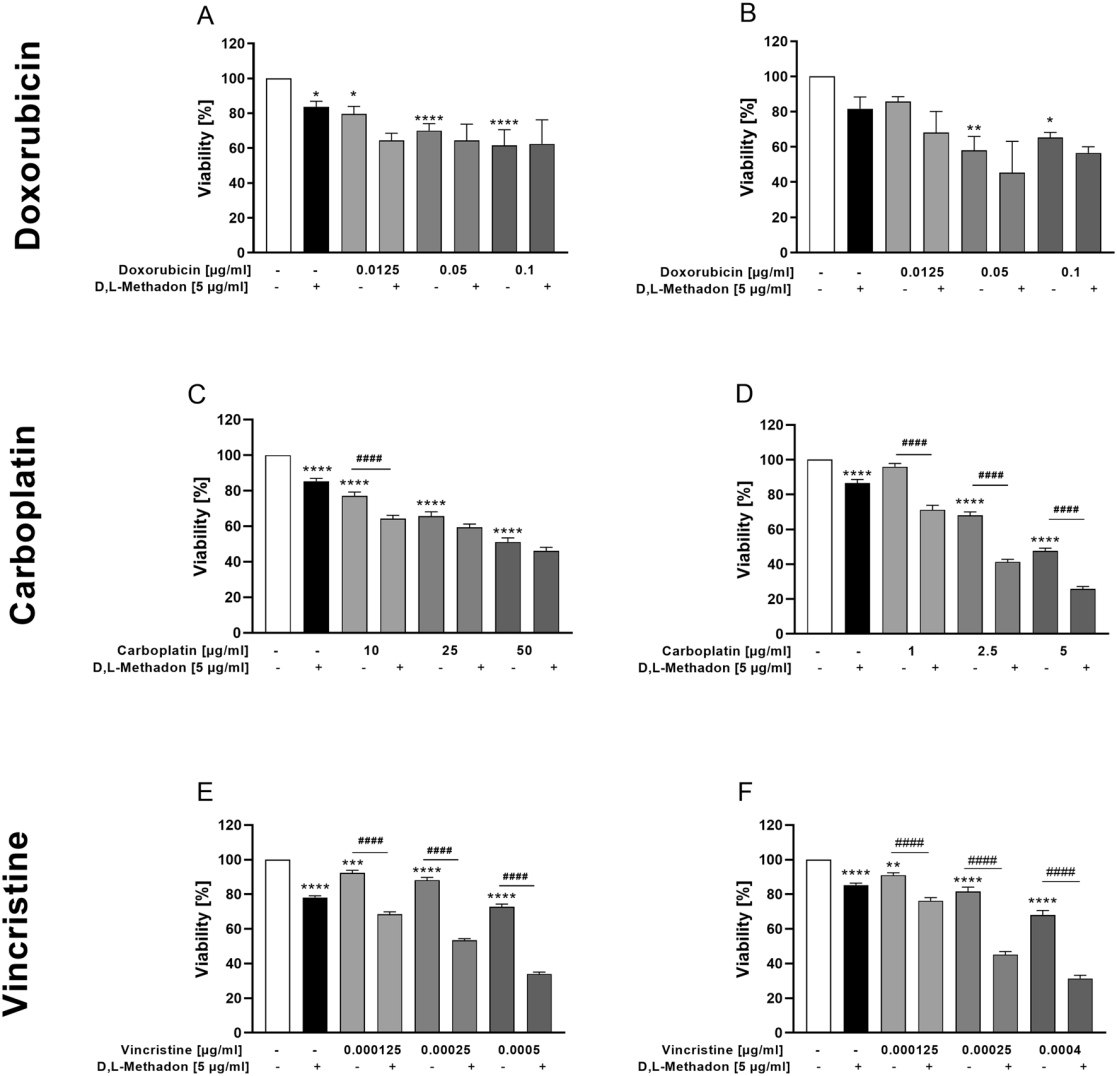
Effects of combined treatment (D,L-methadone/chemotherapeutic agents) on the viability of RMS cells. Arithmetic means ± SEM (*n* = 6) of the relative number of viable RD (left) and RH30 (right) cells after 72 h incubation in the presence of 5 µg/ml D,L-methadone and different concentration of doxorubicin (**A**, **B**), carboplatin (**C**, **D**), and vincristine (**E**, **F**). *(*p* < 0.05), **(*p* < 0.01), ***(*p* < 0.001), ****(*p* < 0.0001) indicate statistical significance to untreated control as well as ^####^(*p* < 0.0001) indicate statistical significance between chemotherapeutic agent alone and in combination with D,L-methadone and cytostatic drug (ANOVA, Bonferroni correction)

Treatment of the RMS cells with doxorubicin in combination with D,L-methadone did not significantly reduce the proportion of viable cells compared to the treatment with doxorubicin alone as shown in Fig. 6(A, B).

Carboplatin in combination with D,L-methadone lead to a significant reduction of cell viability in RH30 cells when compared to the cytostatic drug alone. In RD cells, a significant reduction of cell viability was reached only using 10 µg/ml carboplatin and 5 µg/ml D,L-methadone. Higher concentration of carboplatin did not lead to a further significant reduction of cell viability (Fig. 6 C, D).

The combined treatment of vincristine (independent of concentration) with D,L-methadone resulted in a significant reduction of the proportion of viable cells in both cell lines, compared to the use of the chemotherapeutic drug alone as depicted in Fig. 6 (E, F).

The effects were additive when D,L-methadone was combined with doxorubicin. In RD cells, the combined treatment D,L-methadone/carboplatin had an additive effect, while in RH30 cells the effect was synergistic. A synergistic effect was observed in RH30 and RD cells in combination treatment D,L-methadone/vincristine (Supplementary Fig. 1).

### Effects of combined treatment D,L-methadone and chemotherapy on migration of RMS cells

**Fig. 7:**
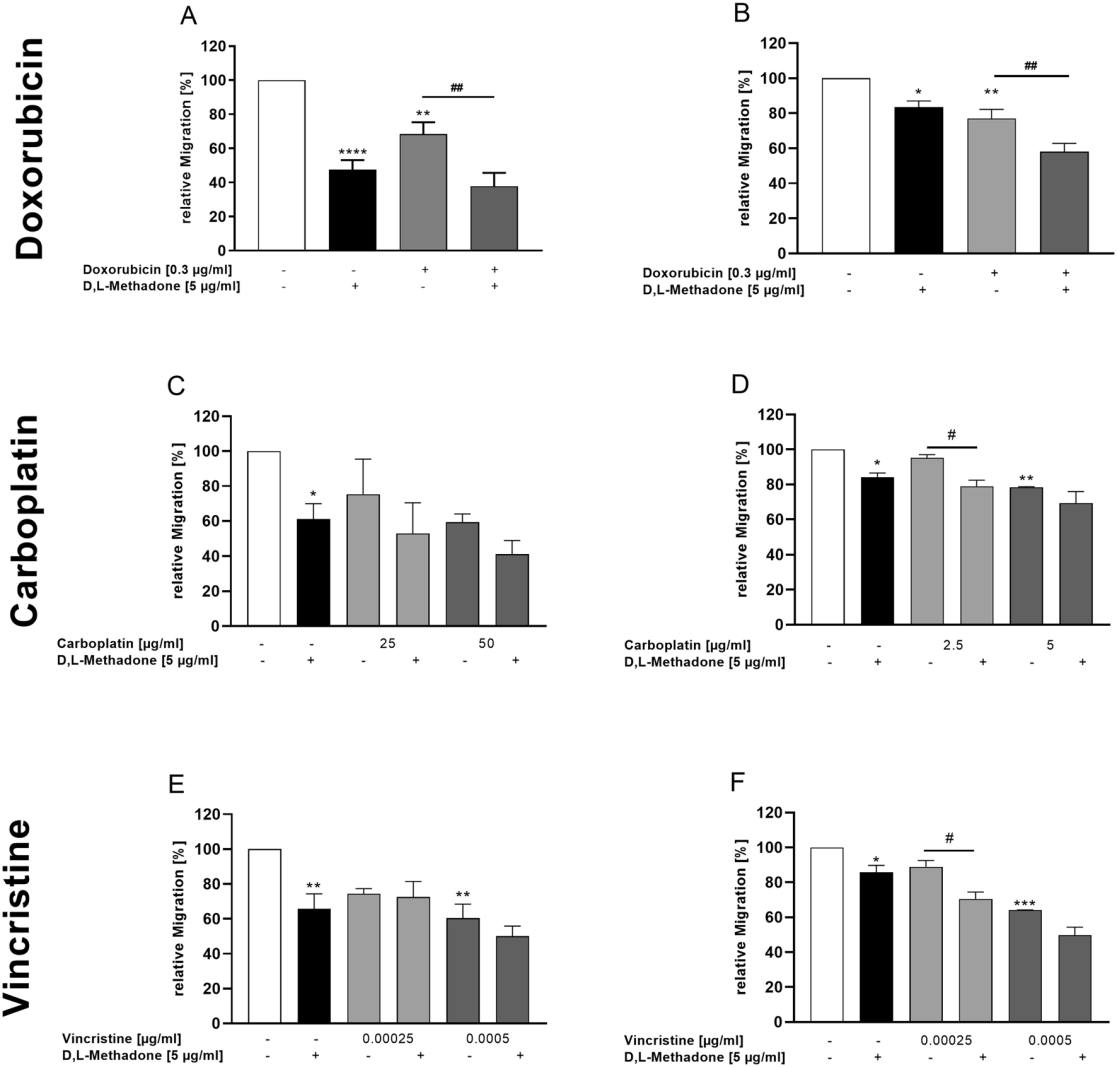
Effects of D,L-methadone in combination with chemotherapeutic drugs on migration of RMS cells. Arithmetic means ± SEM (*n* = 7) of the migration values of RD (left) and RH30 (right) cells before (white bars) and after treatment with different concentrations (grey bars) of doxorubicin (**A**, **B**), carboplatin (**C**, **D**), and vincristine (**E**, **F**) in the presence or absence of 5 µg/ml D,L-methadone (black bars). *(*p* < 0.05), **(*p* < 0.01), ***(*p* < 0.001), ****(*p* < 0.0001) indicates statistically significant difference to untreated control as well as ^#^(*p* < 0.05), ^##^(*p* < 0.01) indicate statistical significance between chemotherapeutic agent alone and in combination with D,L-methadone and cytostatic drug (ANOVA, Bonferroni correction)

We observed that D,L-methadone alone reduced the migration of RMS cells compared to normal muscle cells. We investigated if the migration capacity of RMS cell lines may further be decreased if D,L-methadone is used in combination with the chemotherapy. As shown in Fig. 7, the treatment of cells with doxorubicin in combination with D,L-methadone significantly reduced the migration capacity in both cell lines compared to the treatment with doxorubicin alone, although this effect was more evident in RH30 cells. When used in high concentrations, carboplatin, and vincristine significantly reduce the migration compared to untreated cells. Combined treatment with carboplatin and D,L-methadone as well as vincristine and D,L-methadone significantly reduced migration only in RH30 cells by around 20% and only at concentrations of 2.5 µg/ml carboplatin and 0.00025 µg/ml vincristine.

As shown in the supplementary figure, the combination of D,L-methadone with all three chemotherapy agents had additive effects in almost all combinations. In the RH30 cell line, only the combination with doxorubicin and 0.00025 µg/ml vincristine showed a synergistic effect.

### Effects of combined treatment D,L-methadone/chemotherapy on apoptosis induction and Caspase 3/7 activity in RMS cells

**Fig. 8:**
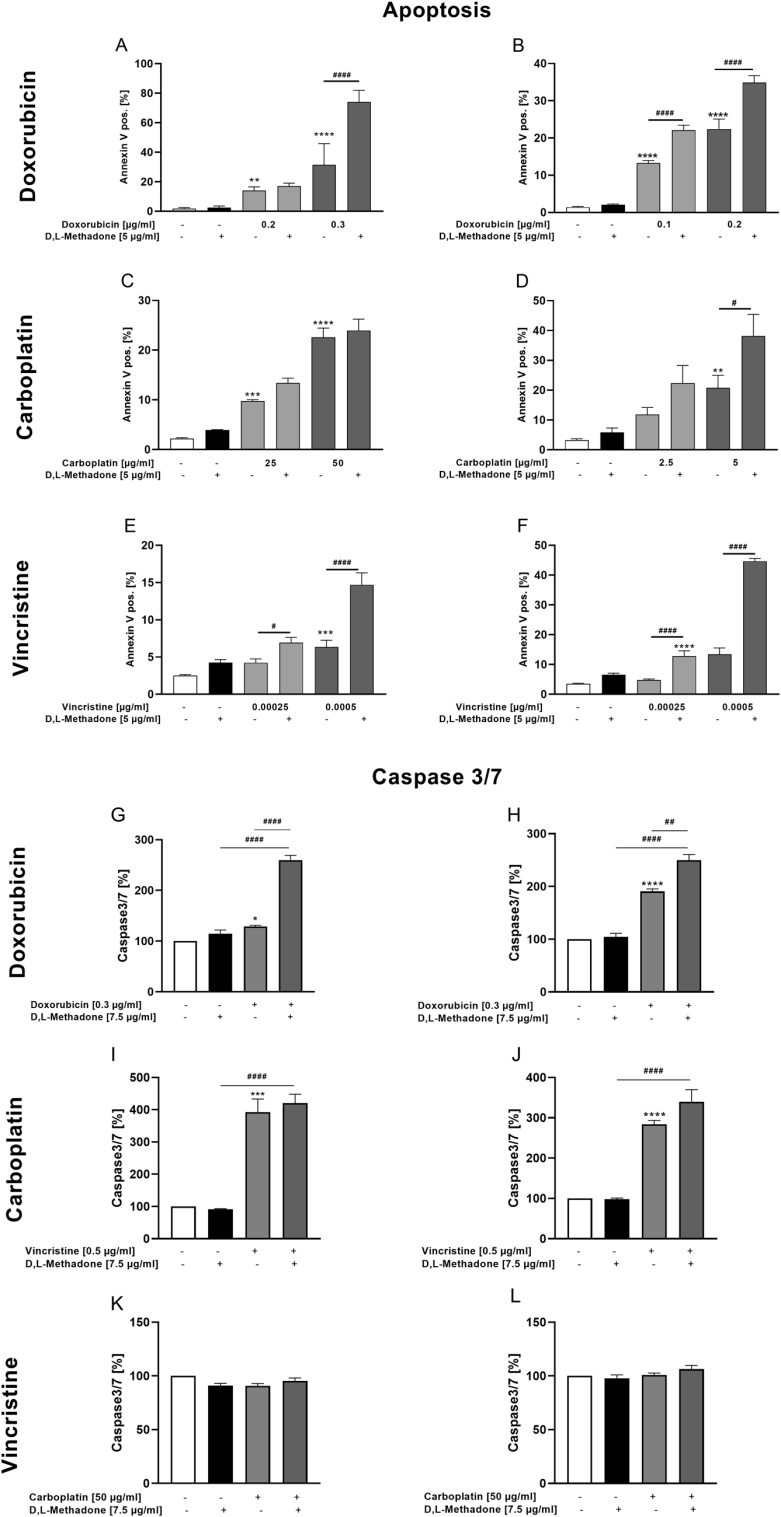
Effects of combined treatment (D,L-methadone/chemotherapy) on induction of apoptosis and Caspase 3/7 activity of RMS cells. Arithmetic means ± SEM (n = 6) of the relative numbers of viable RD (left) and RH30 (right) cells after 72 h incubation with 5 µg/ml D,L-methadone in combination with different concentrations (black bars) of doxorubicin (**A**, **B**), carboplatin (**C**, **D**), and vincristine (**E**, **F**) relative to control (white bars). Caspase 3/7 levels were measured in RD (left) and RH30 (right) cells after 24 h treatment with D,L-methadone (7.5 µg/ml) and doxorubicin (0.3 µg/ml; **G**, **H**), carboplatin (50 µg/ml; **I**, **J**) or vincristine (0.5 µg/ml; **K**, **L**). All data are shown as arithmetic means ± SEM (*n* = 4). *(*p* < 0.01), **(*p* < 0.01), ***(*p* < 0.001), ****(*p* < 0.0001) indicate statistical significance as well as ^#^(*p* < 0.05), ^##^(*p* < 0.01), ^####^(*p* < 0.0001) indicate statistical significance between chemotherapeutic agent alone and in combination with D,L-methadone and cytostatic drug (ANOVA, Bonferroni correction)

The concept of all types of chemotherapeutic drugs is the targeted and simultaneously controlled destruction of tumor cells via apoptosis (Friesen et al. 1996). Therefore, flow cytometry analyses were performed to quantify living, apoptotic, and necrotic cells to gain more differentiated insight into the cytotoxic effects of D,L-methadone. When used in higher concentrations, doxorubicin combined with D,L-methadone lead to a significant increase of the apoptosis rates in both cell lines compared with doxorubicin alone, as shown in Fig. 8 (A, B). This was also evident in the CDI values, which all data show a synergistic effect in both cell lines (Supplementary Fig. 1).

Carboplatin alone induced significant levels of apoptosis in both cell lines, reaching almost 40% of Annexin-V-positive cells in RH30 cell line. The addition of D,L-methadone resulted in a significant increase of the proportion of apoptotic cells only in RH30 cells and only at a concentration of carboplatin of 5 µg/ml (Fig. 8 C, D). As shown in the supplemental figure, the combination of D,L-methadone with carboplatin had additive effects in RD cells and synergistical effects in RH30 cells.

Vincristine in combination with D,L-methadone also determined a significant increase of apoptotic rate in both cell lines compared to the treatment with vincristine alone. In RH30, the combined treatment resulted in a significant increase of apoptotic rate up to 45% compared to treatment with vincristine alone (Fig. 8 E, F). However, a synergistic effect was only seen at the higher concentration of 0.0005 µg/ml vincristine in both cell lines, the lower value showed an additive effect.

To support the evidence that D,L-methadone in combination with chemotherapy activates caspase-dependent apoptosis, caspase-3/7 activity was also examined. We observed that D,L-methadone in combination with doxorubicin significantly and synergistically increased caspase-3/7 activity compared with doxorubicin alone (Fig. 8 G, H). Similar results were also obtained with vincristine as shown in Fig. 8 I, J. Carboplatin had no effect on caspase-3/7 activity neither alone nor in combination with D,L-methadone (Fig. 8K, L). The CDI values showed an additive effect in both RD and RH30 cells (Supplementary Fig. 1).

### Effects of combined treatment D,L-methadone/chemotherapy on ROS production in RMS cells

**Fig. 9:**
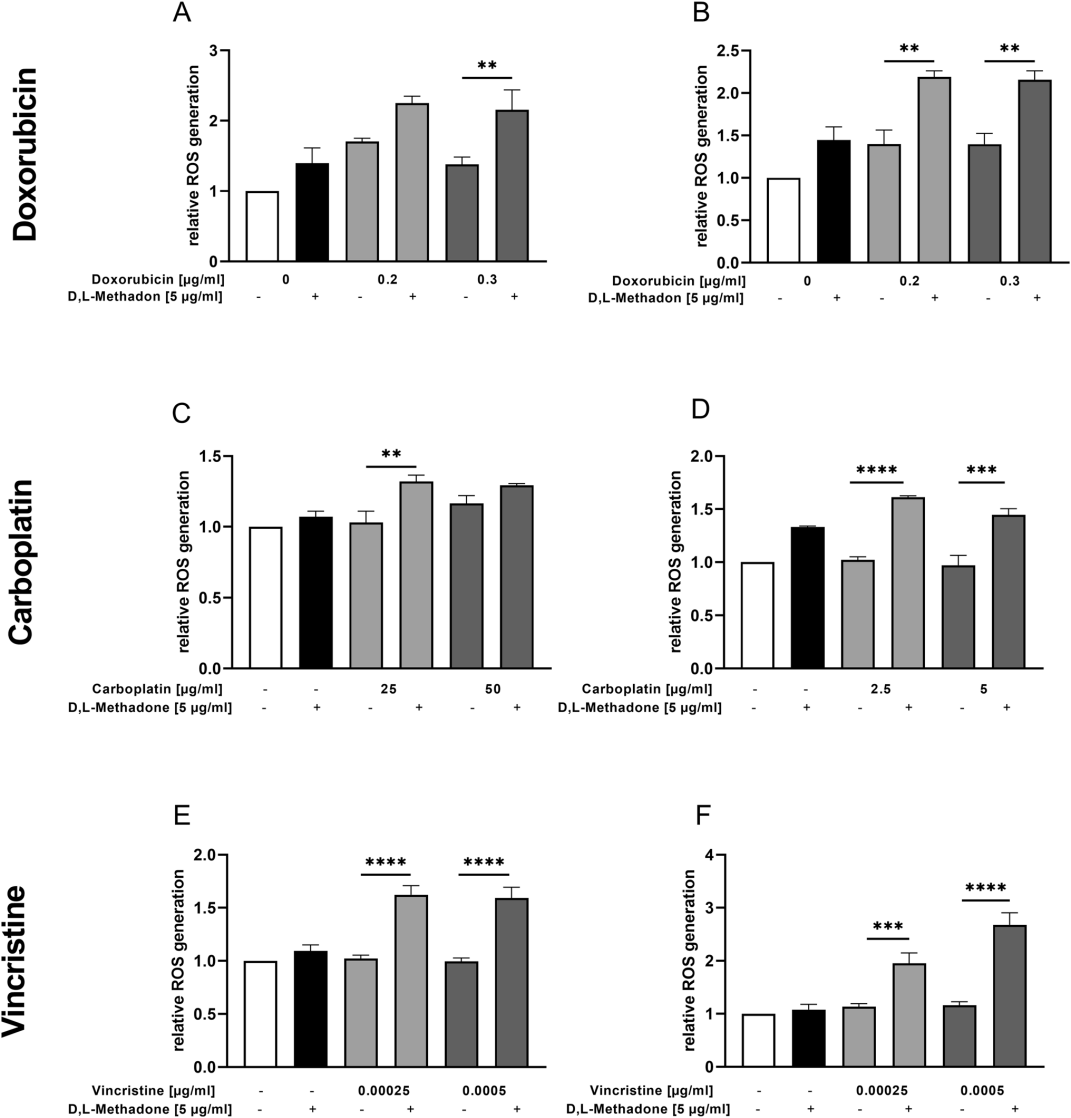
Effects of combined treatment (D,L-methadone/chemotherapy) on production of reactive oxygen species (ROS) by RMS cells. ROS level measurement in RD (left) and RH30 (right) cells after 24 h treatment with D,L-methadone and different concentrations of doxorubicin (**A**, **B**), carboplatin (**C**, **D**), and vincristine (**E**, **F**). Cells were treated with DCFH-DA and measured via flow cytometry. Data are shown as arithmetic means ± SEM (*n* = 6). *(*p* < 0.01), **(*p* < 0.01), ***(*p* < 0.001), ****(*p* < 0.0001) indicate statistical significance (ANOVA, Bonferroni correction)

The production of ROS was evaluated to investigate the mechanisms and pathways which are stimulated using D,L-methadone in combination with cytostatic drugs. We observed that D,L-methadone in combination with doxorubicin had a significant effect on ROS production in both cell lines compared with the treatment with doxorubicin alone, as depicted in Fig. 9 (A, B). The most pronounced effect was encountered in RH30 cells as combined treatment resulted in an increase of ROS production rate by 4–5 times compared to the doxorubicin single treatment. Interestingly, ROS levels remained constant when doxorubicin concentration was increased. The same was true for D,L-methadone. After increase of D,L-methadone concentration from 5 to 7.5 µg/ml, only minimal differences in ROS production in RD and RH30 cells were observed.

The combination treatment of D,L-methadone with carboplatin produced a significant increase of the ROS levels in both cell lines (Fig. 9C, D). In RD cells only, the combination treatment with 25 µg/ml had a significant result on ROS production, in RH30 cells both concentrations (2.5 µg/ml and 5 µg/ml).

The levels of ROS produced by RD and RH30 cells in response to different doses of vincristine combined with D,L-methadone were significantly higher compared to vincristine alone as depicted in Fig. 9 (E, F). As shown in the supplemental figure, the combination of D,L-methadone with doxorubicin, carboplatin, or vincristine had synergistic effects regarding ROS production.

## Discussion

In the present study, we analyzed the effects of D,L-methadone in combination with doxorubicin, carboplatin, and vincristine in pediatric RMS cell lines. We observed that the combined treatment significantly suppressed tumor cell growth and increased ROS production compared to the treatment with cytostatic drugs alone. To our knowledge, this is the first report in the literature addressing the effect of D,L-methadone in combination with cytostatic drugs on pediatric RMS cells.

It has been shown that opioid receptors are expressed by numerous malignancies. Opioid peptides and their receptors were found in primary human breast carcinoma, whereas no presence of opioid system was reported in normal breast tissues (Zagon et al. 1987; Chatikhine et al. 1994; Fichna and Janecka 2004). Several studies have also documented involvement of opioid system in colon cancer (Nylund et al. 2008; Bostwick et al. 1987; Zagon et al. 1987). In our present study, we demonstrated for the first time that the µ-opioid receptor OPRM1 is also present in RMS cells, and that RD cells show significant higher expression levels compared to SkMC.

Certain chemotherapeutic drugs may influence the expression of the OPRM1. Friesen et al. have demonstrated that doxorubicin increases OPRM1 expression in leukemia and glioblastoma cells after 120 h (Friesen et al. 2013, 2014). We found that treatment of RMS cells with doxorubicin has also led to a significant increase of OPRM1 expression in a time-dependent manner. We observed an increase in expression of OPRM1 as early as 12 h after exposure of RMS cells to chemotherapeutic drugs. Similar results were also obtained with vincristine and carboplatin.

In the present study, we demonstrated for the first time that D,L-methadone has a significant cytotoxic effect on RMS cells. We found that treatment with doxorubicin led to a reduction of cell viability with significant increase of apoptosis rates, and this effect was further significantly enhanced by additional treatment with D,L-methadone. Preclinical evidence has shown that D,L-methadone increases the sensitivity towards chemotherapy of different types of cancer cells (Friesen et al. 2008, 2014; Landgraf et al. 2019; Kaina et al. 2020; Haas et al. 2021). Landgraf et al. have reported that D,L-methadone significantly enhanced the toxicity of doxorubicin and cisplatin in HLaC78 cells (larynx carcinoma) (Landgraf et al. 2019). Stadlbauer et al. have also found that co-treatment of doxorubicin with L-methadone determines a dose-dependent increase in the apoptosis rate up to 88% in PC-3 cells (prostate cancer) (Stadlbauer et al. 2017). Friesen et al. demonstrated that the treatment with doxorubicin and D,L-methadone has led to an increase of apoptosis rate by factor 4 in leukemia cells and by factor 8 in glioblastoma cells. Our measurements showed a significant increase of apoptosis rates in RMS cells after treatment with 5 µg/ml D,L-methadone for 72 h. However, an essential feature of leukemia cell lines is the reaction of cells with apoptosis induction to D,L-methadone also as a single therapy. In RMS cells, D,L-methadone alone did not lead to an increase in apoptosis. Nevertheless, in the cell viability analyses, we observed a significant reduction of cell viability in RMS cells. It may be concluded that D,L-methadone leads to an inhibition of cellular metabolism but does not have its own mechanisms that could guide the cell into apoptosis independently of chemotherapeutic drugs.

It has been reported by Patel et al. on macrophages that activation of µ-opioid receptor leads to an inhibition of adhesion and subsequently to an inhibition of cell migration (Patel et al. 2003). Our results are in line with these findings. We found that D,L-methadone in combination with cytostatic drugs significantly inhibited cell migration compared to the chemotherapeutic drugs alone.

For doxorubicin, it has already been shown that the reorganization of actin influences the cytoskeletal stability of the cell and that doxorubicin has an effect on the migration behavior of the cells. Croft et al. have demonstrated that genotoxic stress and increased ROS formation during doxorubicin treatment influence Rho-dependent signaling pathways (Croft et al. 2011). Rho-GTPases are key proteins which are responsible for the regulation and dynamics of the actin cytoskeleton of migrating cells, and are particularly involved in tumor metastasis and angiogenesis (Ridley 2015). Regarding D,L-methadone, interactions with ion channels and the associated modulation of the transmembrane potential may also play a role. Activation of the µ-opioid receptor results in activation of the Kir3 channel with outflow of potassium cations and inhibition of T, L-, and N-type calcium channels with reduction of transmembrane calcium flow (Seseña et al. 2014; Al-Hasani and Bruchas 2011). Directed migration is based on local calcium currents that determine a cytoplasmic calcium gradient within the cell and subsequently affect different calcium-effector proteins that regulate the activity of cytoskeletal proteins (Wei et al. 2012). The interaction of D,L-methadone with these calcium channels influence the transmembrane calcium flow and represent a possible mechanism through which the effect of doxorubicin is enhanced and tumor cell migration is inhibited. This might have consequences on metastasization, for which migration and adhesion are important factors.

Modes of cell demise comprise a number of processes such as apoptosis and necrosis, and intermediate options (Perez-Alvarez et al. 2010). Besides calcium, ROS have also been proposed as second messengers involved in the initial steps of cellular death (Perez-Alvarez et al. 2010; Leist and Jäättelä 2001). In the present work, we could demonstrate that D,L-methadone combined with chemotherapeutic drugs significantly increased the production of ROS in RMS cells. We observed that ROS levels remained constant when doxorubicin concentration was increased. Similarly, the increase of D,L-methadone concentration determined only minimal differences in ROS production in RD and RH30 cells. This suggests that both doxorubicin and D,L-methadone alone induce a baseline rate of ROS, but this rate cannot be further increased using higher concentrations of the respective drug. In contrast, the combination treatment allows a significant increase of ROS production which can be up to 4–5 times higher as observed in RH30 cells, suggesting a synergistic effect.

ROS increases the permeability of the outer mitochondrial membrane and thus initiates the activation of caspase cascade through the release of cytochrome C, ultimately leading to cell death (Ryter et al. 2007). However, recent evidence shows that ROS also exerts its cytotoxic effects by influencing signaling pathways involved in extrinsic apoptosis. For example, Zhang et al. demonstrated that elevated ROS levels lead to the formation of CD95/TNFα receptor clusters, and, through these, to the initiation of extrinsic apoptosis signaling pathways in coronary arterial endothelial cells (Zhang et al. 2007, 2006).

Our observation in the present study, that D,L-methadone combined with chemotherapeutic drugs leads to an increased ROS production is in concordance with recent findings: the µ-opioid receptor is related not only to the classical cAMP protein kinase signaling pathway, but also influences a large number of cellular processes such as formation of ROS mediated by β-arrestin and phospholipase D2 (PLD2) (Koch et al. 2009; Saify and Saadat 2015). Koch et al. demonstrated that receptor internalizing agonists such as D,L-methadone determine a strong induction of NADH /NADPH oxidase-mediated ROS synthesis via PLD2 (Koch et al. 2009; Williams et al. 2013). These data explain the formation of ROS under single treatment with D,L-methadone and the observed enhancement of ROS production after combined treatment. The mechanisms by which D,L-methadone and chemotherapeutic drugs induce apoptosis likely depend on the cancer cell type. In our study, we observed that the induction of apoptosis occurs through up-regulation of caspase-3/7 activation and increased ROS production.

In the CDI analysis, we were able to demonstrate that the effect of D,L-methadone in combination with chemotherapy drugs was additive or synergistic depending on the assay performed. In summary, the sum of most effects was synergistic. However, there were also exceptions. For example, the sum of effects on migration was additive regardless the chemotherapy drug used. When looking at cell viability, the effects were of D,L-methadone combined with doxorubicin were additive. The effects of the combined treatment D,L-methadone/carboplatin were also additive regarding apoptosis and caspase-3/7 activation in RD cells.

There are certain limitations of the study. All investigations were performed in vitro. Therefore, it is also difficult to conclude whether in vivo the effects are synergistic or additive. Therefore, further in vivo studies should be performed to conclusively answer this question. Furthermore, underlying molecular mechanisms could not be observed extensively. However, this was the first systematical analysis so far. Therefore, further investigations including in vivo analyses seem justified.

In conclusion, D,L-methadone proves to enhance the cytotoxic activity of standard chemotherapeutic drugs on RMS in vitro. This study may provide the foundation for new therapeutic strategies establishing D,L-methadone as an additional therapeutic drug in the treatment of children with advanced RMS.

## Supplementary Information

Below is the link to the electronic supplementary material.

Supplementary file1 Fig.1 Coefficients of drug interaction (CDI) according to Bliss Indepencence. CDI values of viability assay (A-C), migration (E-G), induction of apoptosis (H-J), caspase 3/7 activity (K-M), and ROS production (N-P). Values are related to the combination with D,L methadone in combination with the chemotherapeutic agents doxorubicin (A, E, H, K, N), carboplatin (B, F, I, L, O) and vincristine (C, G, J, M, P). Values below 1 (broken line) indicate synergism, values above 1 indicate antagonism, 1 means additive effects. (TIF 1600 KB)

Supplementary file2 (PDF 2000 KB)

Supplementary file3 (PNG 56 KB)

Supplementary file4 (PNG 39 KB)
