# Supplementary figures and images for: D,L-Methadone enhances the cytotoxic activity of standard chemotherapeutic agents on pediatric rhabdomyosarcoma
Source: J Cancer Res Clin Oncol. 2022 Feb 19;148(6):1337–50. doi: 10.1007/s00432-022-03945-y (PMC9114081; doi:10.1007/s00432-022-03945-y)

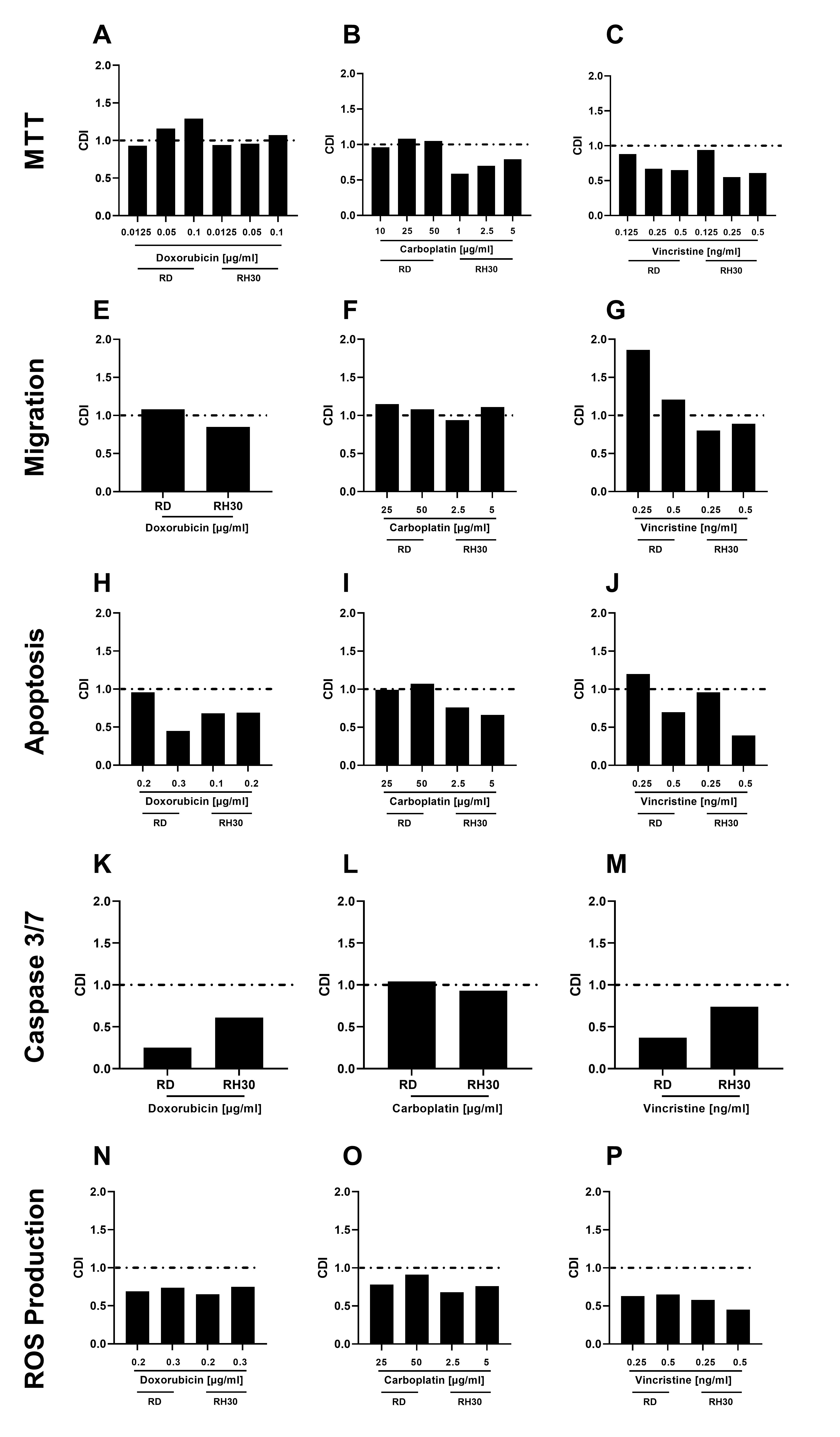

Supplement: Supplementary file 1 — Supplementary file1 Fig.1 Coefficients of drug interaction (CDI) according to Bliss Indepencence. CDI values of viability assay (A-C), migration (E-G), induction of apoptosis (H-J), caspase 3/7 activity (K-M), and ROS production (N-P). Values are related to the combination with D,L methadone in combination with the chemotherapeutic agents doxorubicin (A, E, H, K, N), carboplatin (B, F, I, L, O) and vincristine (C, G, J, M, P). Values below 1 (broken line) indicate synergism, values above 1 indicate antagonism, 1 means additive effects. (TIF 1600 KB) [file 432_2022_3945_MOESM1_ESM.tif]

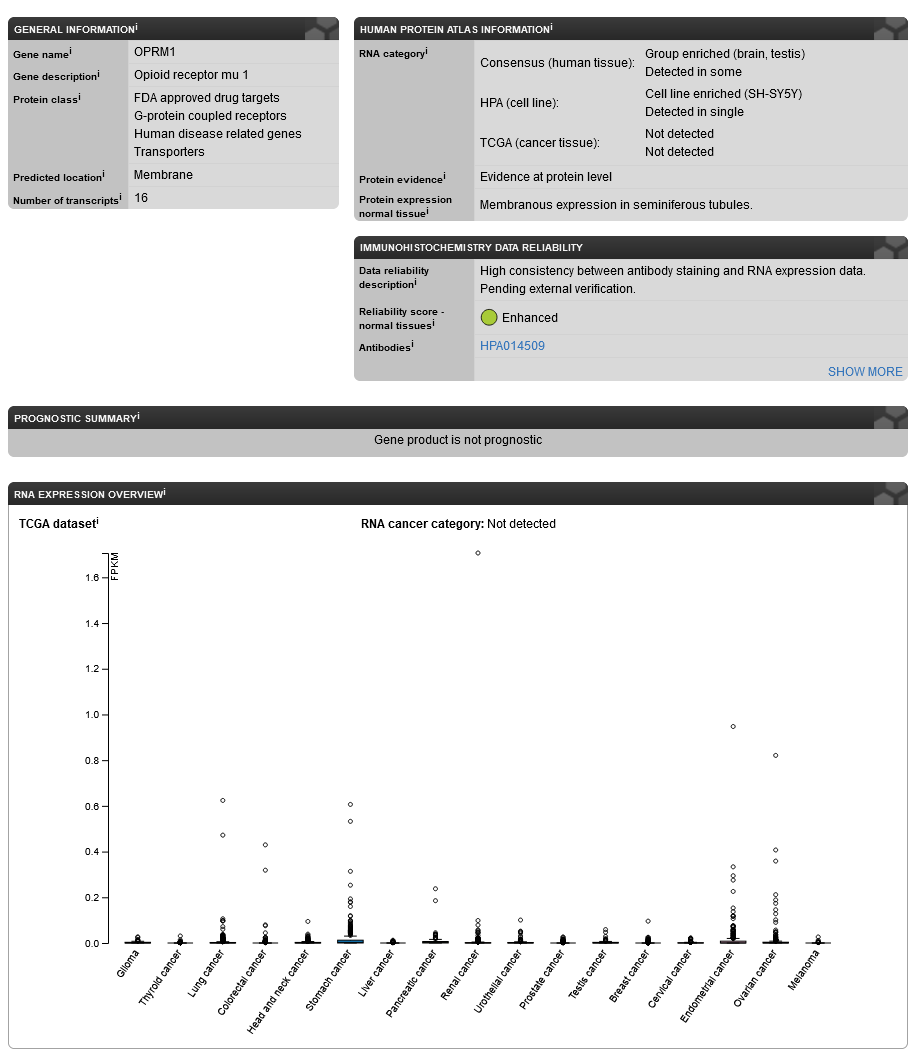

Supplement: Supplementary file 3 — Supplementary file3 (PNG 56 KB) [file 432_2022_3945_MOESM3_ESM.png]

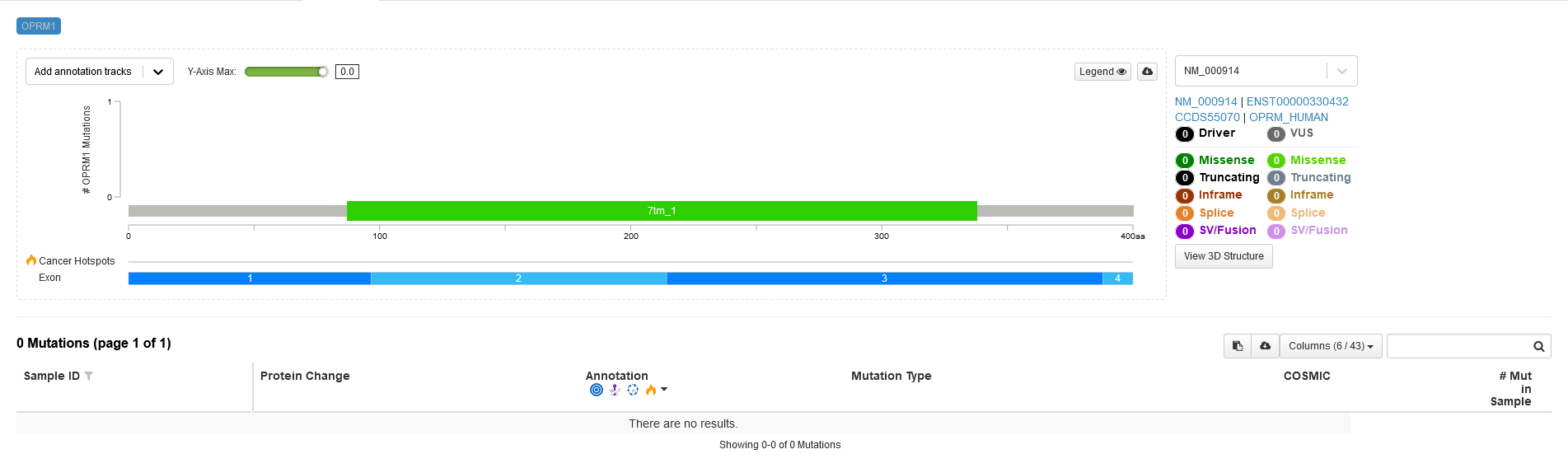

Supplement: Supplementary file 4 — Supplementary file4 (PNG 39 KB) [file 432_2022_3945_MOESM4_ESM.png]
