# Supplementary material for: D,L-Methadone enhances the cytotoxic activity of standard chemotherapeutic agents on pediatric rhabdomyosarcoma
Source: J Cancer Res Clin Oncol. 2022 Feb 19;148(6):1337–50. doi: 10.1007/s00432-022-03945-y (PMC9114081; doi:10.1007/s00432-022-03945-y)

Figure 1

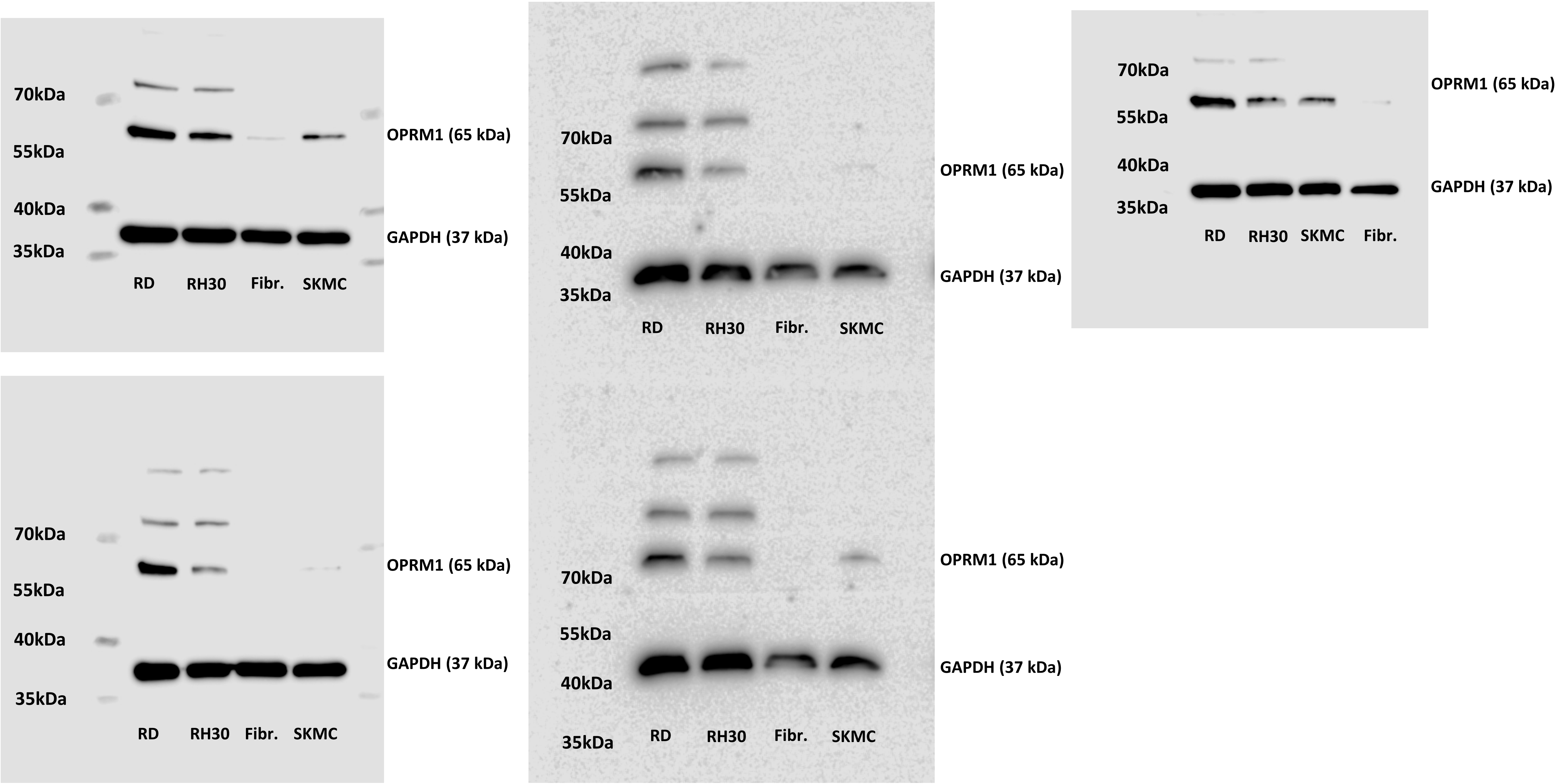
70kDa
55kDa
40kDa
35kDa
OPRM1 (65 kDa)
GAPDH (37 kDa)
RD
RH30
Fibr.
SKMC
70kDa
55kDa
40kDa
35kDa
OPRM1 (65 kDa)
GAPDH (37 kDa)
RD
RH30
Fibr.
SKMC
70kDa
55kDa
40kDa
35kDa
OPRM1 (65 kDa)
GAPDH (37 kDa)
RD
RH30
Fibr.
SKMC
70kDa
55kDa
40kDa
35kDa
OPRM1 (65 kDa)
GAPDH (37 kDa)
RD
RH30
Fibr.
SKMC
70kDa
55kDa
40kDa
35kDa
OPRM1 (65 kDa)
GAPDH (37 kDa)
RD
RH30
SKMC
Fibr.

Figure 5 – RD Doxorubicin

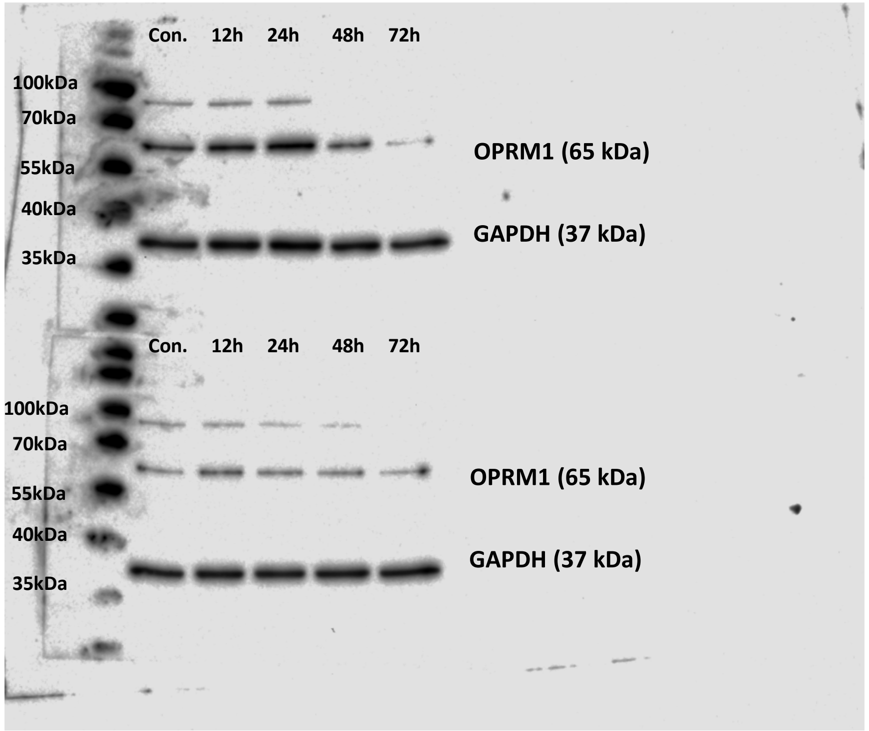

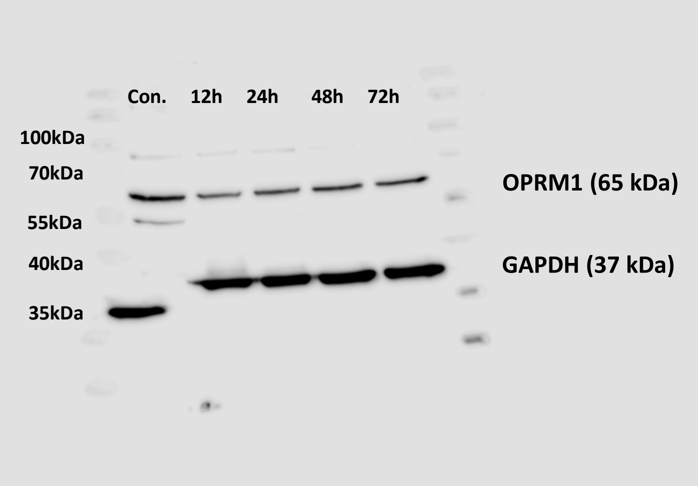

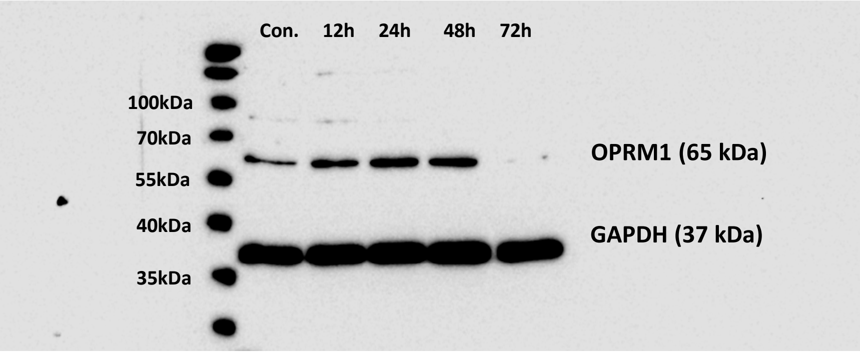

Figure 5 – RH30

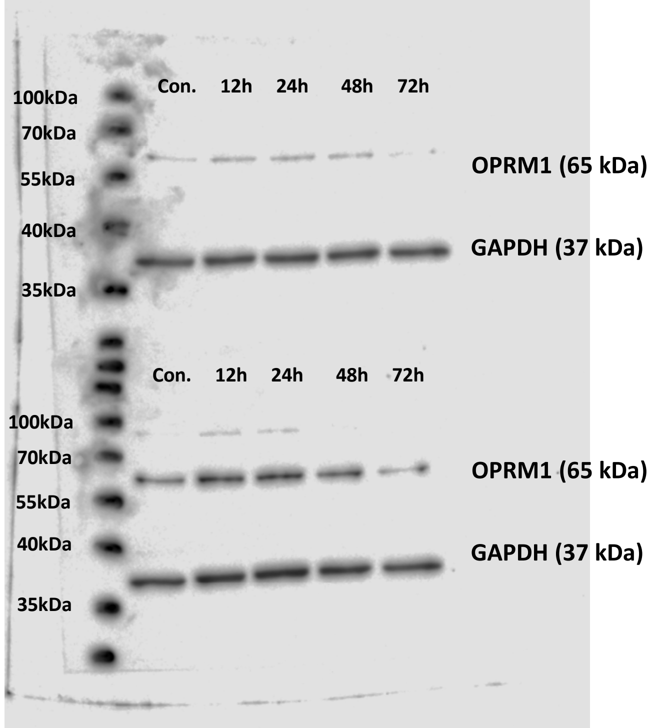

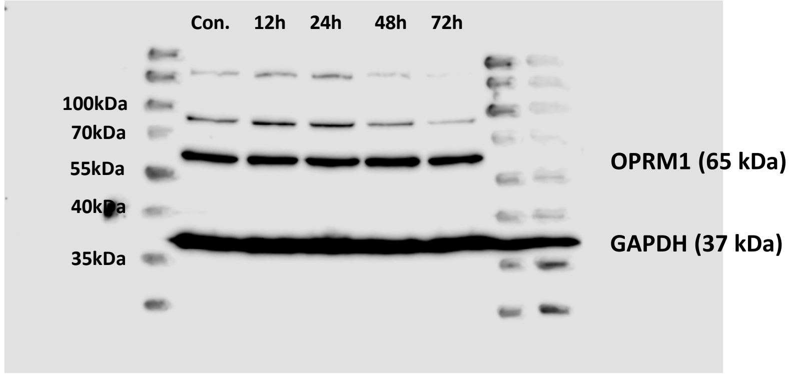

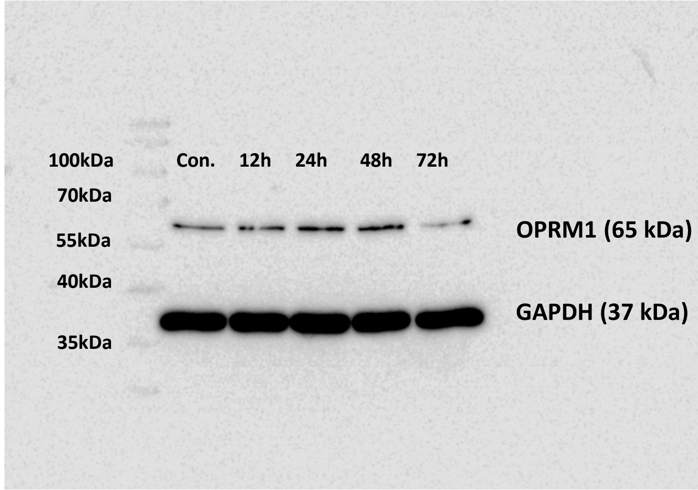

Figure 5 – RH30 – Vincristin & Carboplatin

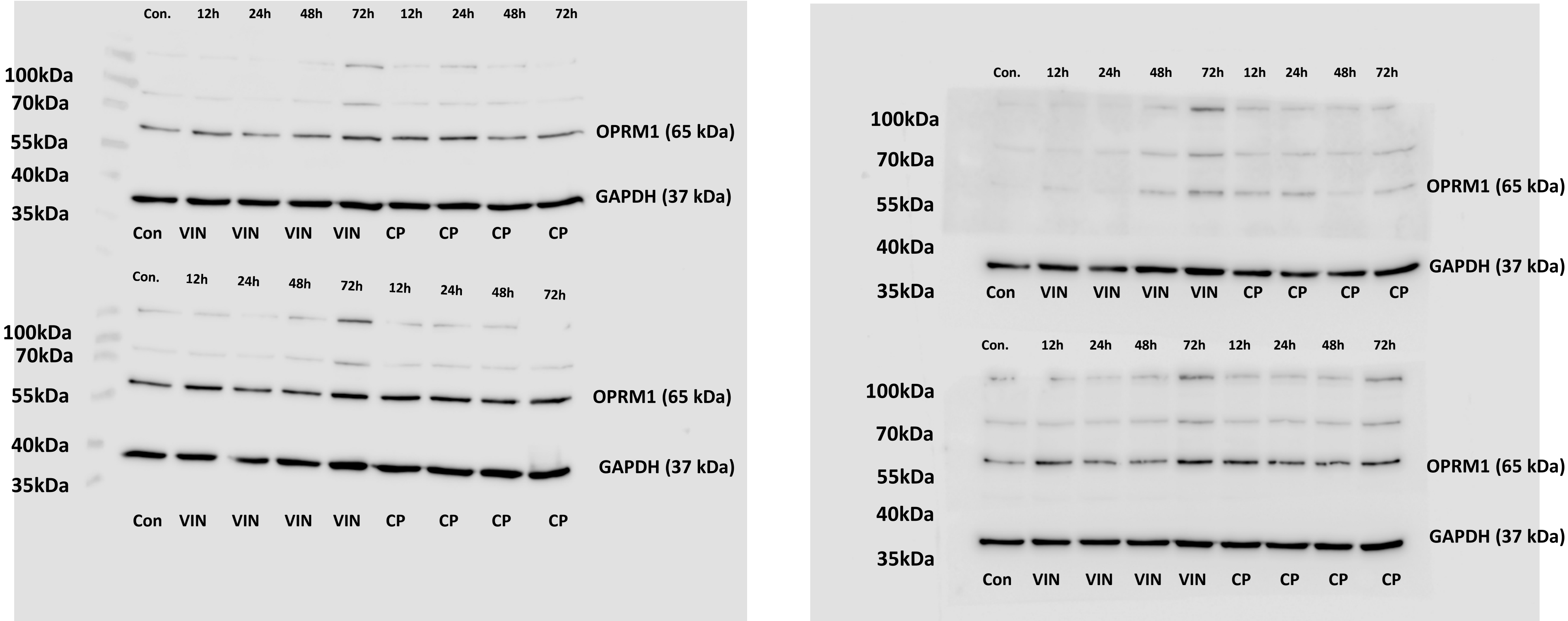

Figure 5 – RD – Vincristin & Carboplatin

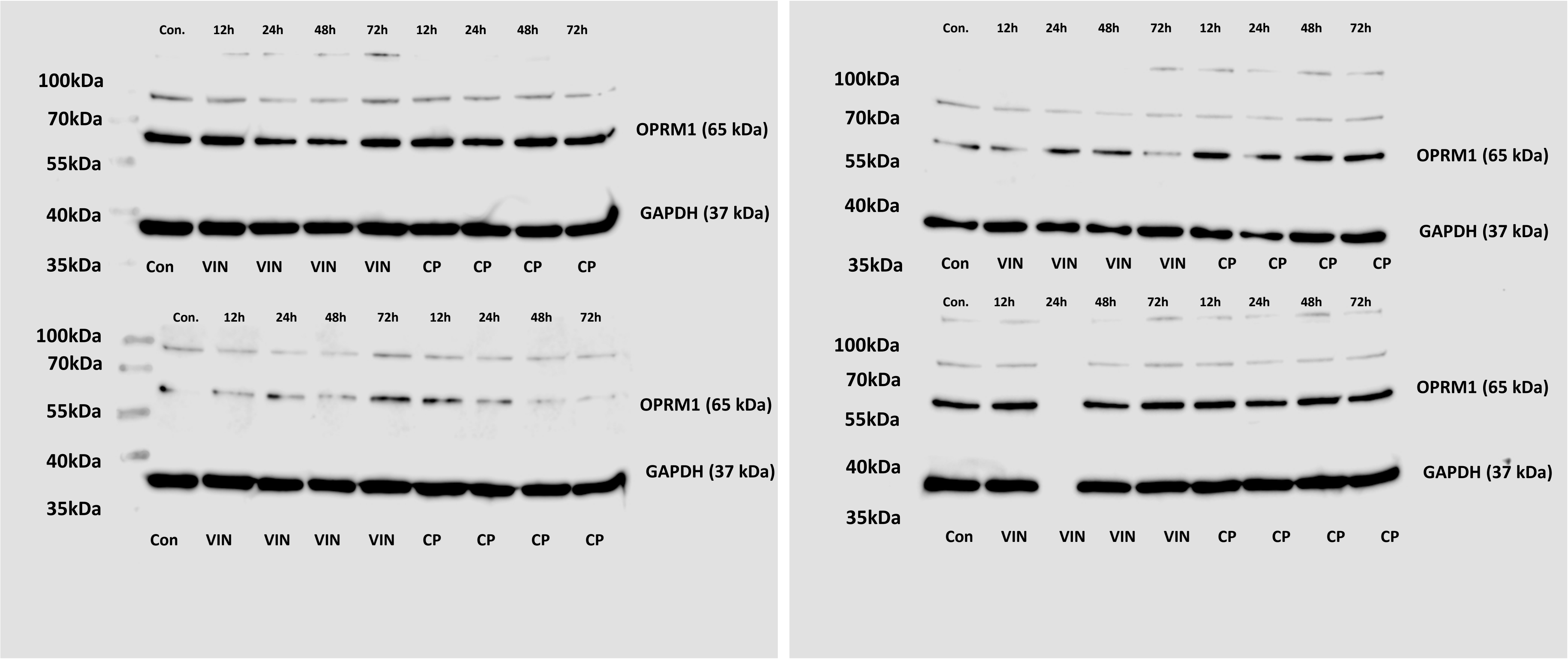

Supplement: Supplementary file 2 — Supplementary file2 (PDF 2000 KB) [file 432_2022_3945_MOESM2_ESM.pdf]
